# Neural parameter calibration for dengue outbreak forecasting

**DOI:** 10.1371/journal.pone.0339398

**Published:** 2026-02-25

**Authors:** Hoang Viet Pham, Khuong Trung Dang Nguyen, Thirumalaisamy P. Velavan, Khanh Duc Tran

**Affiliations:** 1 Faculty of Engineering, Vietnamese-German University, Ho Chi Minh City, Vietnam; 2 Institute of Tropical Medicine, University of Tuebingen, Tuebingen, Germany; 3 Vietnamese German Center for Medical Research (VG-CARE), Hanoi, Vietnam; 4 Faculty of Medicine, Duy Tan University, Da Nang, Vietnam; Centers for Disease Control and Prevention, UNITED STATES OF AMERICA

## Abstract

Dengue fever poses a growing public health challenge in tropical and subtropical regions, with transmission driven by complex interactions among viral and host. Computational models, often expressed as ordinary differential equations (ODEs), are widely used to understand complex systems such as dengue fever transmission dynamics. However, traditional parameter estimation methods such as Markov chain Monte Carlo (MCMC) often require complex setups and are computationally expensive. In this study, we choose a compartmental model extended to human and mosquito populations, estimate its parameters using neural parameter calibration (NPC), and validate the approach using datasets collected from South America and Southeast Asia. The extended compartment model (ECM) is expressed using seven ODEs, describing dengue transmission dynamics between humans and mosquitoes. NPC involves using a neural network to learn the posterior distribution of parameters and initial conditions of the model in consideration. We analyzed six surveillance datasets on cumulative dengue cases, comprising data from three cities (Bello, Iquitos, and San Juan) and three Southeast Asian countries (Vietnam, the Philippines, and Cambodia). NPC achieved significantly faster run times than MCMC: 408 seconds on average versus 2616.01 seconds for city-level analyses and 368 seconds on average versus 2998.83 seconds for country-level analyses. Meanwhile, it delivers comparable accuracy: mean squared error (MSE) 0.00678 versus 0.01638 for the city datasets; and 0.00605 versus 0.01897 for the country datasets. The experimental results demonstrate that combining ECM with NPC enables accurate dengue outbreak forecasts at substantially lower computational cost, offering a practical tool that supports timely response, especially in low-resource environments such as Southeast Asia.

## 1 Introduction

Dengue fever, an expanding mosquito-borne disease in tropical and subtropical regions, threatens half of the world’s population across 129 countries, causing an estimated 100-400 million infections annually, with mortality up to 20% in severe cases without prompt treatment [1–4]. Recurrent large outbreaks are expected to result in a cumulative economic burden of approximately $306 billion by 2050 [5]. Dengue transmission dynamics arise from complex interactions among viral replication cycles, environmental conditions (e.g. temperature, rainfall, humidity) [6–8], and human factors (e.g. population movement, immunity profiles) [9,10]. These complexities highlight the need for computational models capable of forecasting outbreaks accurately and efficiently to support early warning and timely intervention.

Computational models, often expressed through systems of ordinary differential equations (ODEs), are widely used to describe the evolution of multiple dependent variables using derivatives with respect to a single independent variable. Specifically, compartmental models, ODE systems that track the rates of change in the susceptible, exposed, infectious, and recovered compartments (SEIR), form the backbone of mathematical epidemiology. They integrate demographic data and are calibrated against parameters to simulate and forecast outbreak dynamics [11,12]. Although traditional compartmental models provide flexibility in adapting to diverse epidemiological contexts and retain interpretability through clear mechanistic representation, they still have notable limitations. They are mostly limited to a single component, human only, and often require considerable time and resources for parameter estimation [13].

Recent research on data-driven parameterization of computational models for uncertainty quantification has explored various directions, including hierarchical, nonparametric, ensemble, and Bayesian methods [14,15]. Among these, Bayesian techniques, such as Markov chain Monte Carlo (MCMC), Hamiltonian Monte Carlo, and Langevin dynamics-based samplers, like the Metropolis-adjusted Langevin algorithm and its variants [16], are increasingly used. These techniques have been used to capture both the dynamics of viral transmission and the uncertainties arising from parameter variability by running multiple simulations and analyzing the resulting statistical distributions [17,18]. However, Bayesian parameter estimation and uncertainty quantification generally rely on posterior sampling, which faces three key computational challenges. First, the complex a priori setup of initial conditions and parameter ranges creates substantial implementation barriers. Second, when likelihood evaluations require numerical integration of ODEs, high-dimensional estimation becomes expensive, this leads gradient-driven MCMC methods to exhibit random-walk behavior, resulting in excessive computational time and resource, that makes inference impractical during an outbreak. Third, burn-in periods and sample rejections further waste computational resources. This persistent trade-off between computational efficiency and sampling accuracy in Bayesian ODE parameter estimation underscores the need for alternative approaches such as neural parameter calibration (NPC).

Neural parameter calibration (NPC) is an approach that uses a neural network to estimate model parameters and initial conditions so the model outputs closely match real-world observations. NPC overcomes the computational limitations of traditional Bayesian methods by using a neural network to estimate posterior distributions over parameters for large-scale multi-agent models, achieving two- to three-orders-of-magnitude speedups with improved accuracy compared to classical Bayesian techniques [19]. In COVID-19 applications, a neural network method was used to calibrate the parameters and initial conditions of an ODE model, which simulated the spread of the virus in Berlin in 2020 [20]. Despite its promise for epidemic modeling, NPC has not yet been adapted for dengue fever modeling, suggesting an interesting line of research: applying fast NPC to dengue outbreak prediction models.

This study addresses the aforementioned key challenges by pursuing several objectives. We employ an extended compartmental model (ECM), which comprises human and mosquito components and is formulated using seven ODEs to describe dengue transmission dynamics. The model explicitly represents the SEIR states for humans alongside the Susceptible, Exposed, and Infectious states for mosquitoes. We then apply NPC, which uses a neural network to estimate posterior distributions of parameters and initial conditions of the ECM. We conduct parameter uncertainty quantification and basic reproductive number sensitivity analysis. We run NPC on six surveillance datasets of cumulative dengue cases from three cities (Bello, Iquitos, San Juan) and three Southeast Asian countries (Vietnam, the Philippines, Cambodia). Finally, we perform an analysis between NPC and MCMC to show the viability of NPC against state-of-the-art Bayesian methods.

The article is organized as follows. Sect 2 details the ECM, which includes the equations governing the transmission dynamics of dengue between the mosquito and the human population. It also introduces the NPC and MCMC parameter estimation frameworks for the ECM, and presents the calibration and projection datasets. Sect 3 describes the experimental setup for comparing the NPC and MCMC frameworks. Sect 4 evaluates the performance of the neural calibration method against MCMC benchmarks, comparing computational efficiency, predictive accuracy, uncertainty quantification, and sensitivity analysis for the basic reproductive number (*R*_0_) in the analysis for both city and country datasets. Finally, Sect 5 discusses the results, including key findings, limitations, and future directions.

## 2 Methodologies

### 2.1 Modeling dengue transmission dynamics

**Fig 1:**
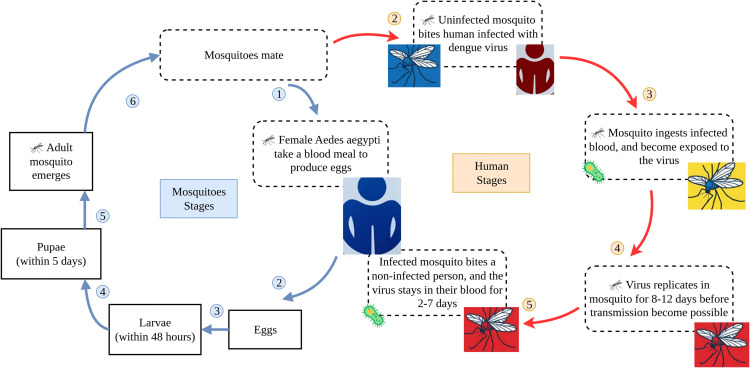
Human-mosquito transmission dynamics of dengue fever.

Fig 1 illustrates the mosquito-borne virus transmission cycle between Aedes aegypti mosquitoes and human hosts. The cycle begins when an infectious female mosquito bites a healthy human for blood meal, which is essential for egg production and maturation. During this feeding process, the mosquito transfers the saliva-containing virus to the human host, initiating infection. When sufficient viremia is established, known as the intrinsic incubation period, human-to-mosquito transmission becomes possible. Viremic individuals may recover within 1-2 weeks of symptom onset, depending on illness severity. When a susceptible mosquito subsequently feeds on this viremic human, it ingests the virus along with the blood meal. Within the mosquito, the virus must overcome several anatomical barriers, first infecting and replicating in cells before spreading through the salivary glands. The process, known as the extrinsic incubation period, typically takes 8-12 days to complete, after which mosquito-to-human transmission becomes possible. Once an infectious adult mosquito emerges, it seeks another blood meal, transmitting the virus to a new human host and continuing the transmission cycle [21]. The mosquito progresses through its entire life cycle, developing from eggs laid in water containers to larvae (within 48 hours), then pupae (within 5 days) and finally emerging as adult mosquitoes. The entire mosquito development cycle from egg to adult takes approximately 8-10 days under optimal conditions.

**Fig 2:**
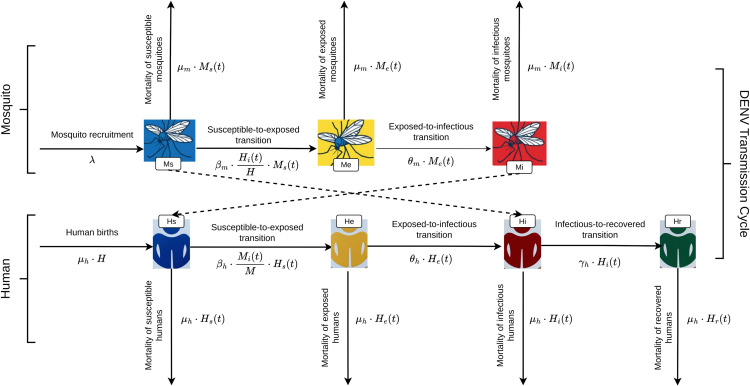
Compartmental model structure for dengue transmission dynamics.

To capture human-mosquito transmission dynamics, we adapt the model formulation of [22], following a compartmental structure that separates mosquitoes (vector) and humans (host) components. This framework converts the biological processes, described above, into measurable parameters that control how the population moves between different states, as visually summarized in the flow diagram Fig 2.

Dengue transmission begins when a susceptible female mosquito *M*_*s*_(*t*)(*t* denotes the dependence on time) bites an infectious human *H*_*i*_(*t*), turning the mosquito into an exposed carrier *M*_*e*_(*t*). The rate at which the turning process occurs, denoted $\beta_{m}\frac{H_{i}(t)}{H}M_{s}(t)$, depends on two factors: (a) the product of the transmission rate from humans to mosquitoes $\beta_{m}$ and the number of susceptible mosquitoes *M*_*s*_(*t*); and (b) the proportion of infectious humans in the population $\frac{H_{i}(t)}{H}$. Reflecting the biological extrinsic incubation period described earlier, exposed mosquitoes become infectious *M*_*i*_(*t*) at a rate $\theta_{m}$. Susceptible humans *H*_*s*_(*t*) become exposed *H*_*e*_(*t*) through infectious mosquito bites at a rate $\beta_{h}\frac{M_{i}(t)}{M(t)}H_{s}(t)$. Here, $\beta_{h}$ quantifies the transmission rate from the mosquito to the human, while $\frac{M_{i}(t)}{M(t)}$ represents the proportion of infectious mosquitoes in the population. Following the intrinsic incubation period described earlier, an exposed human *M*_*e*_(*t*) becomes an infectious state *M*_*i*_(*t*) at a rate $\theta_{h}$ and subsequently recovers *H*_*r*_(*t*) at a rate $\gamma_{h}$. The model also includes natural mosquito mortality rates $\mu_{m}$ and a constant human birth and mortality rate $\mu_{h}$.

The mathematical representation of the human-mosquito transmission dynamics is given by the following ODEs system Eq (1):

$$\frac{dM_{s}(t)}{dt}=\lambda -\beta_{m}\cdot \frac{H_{i}(t)}{H}\cdot M_{s}(t)-\mu_{m}\cdot M_{s}(t)\;\frac{dM_{e}(t)}{dt}=\beta_{m}\cdot \frac{H_{i}(t)}{H}\cdot M_{s}(t)-(\theta_{m}+\mu_{m})\cdot M_{e}(t)\;\frac{dM_{i}(t)}{dt}=\theta_{m}\cdot M_{e}(t)-\mu_{m}\cdot M_{i}(t)\;\frac{dH_{s}(t)}{dt}=\mu_{h}\cdot H-\beta_{h}\cdot \frac{M_{i}(t)}{M(t)}\cdot H_{s}(t)-\mu_{h}\cdot H_{s}(t)\;\frac{dH_{e}(t)}{dt}=\beta_{h}\cdot \frac{M_{i}(t)}{M(t)}\cdot H_{s}(t)-(\theta_{h}+\mu_{h})\cdot H_{e}(t)\;\frac{dH_{i}(t)}{dt}=\theta_{h}\cdot H_{e}(t)-(\gamma_{h}+\mu_{h})\cdot H_{i}(t)\;\frac{dH_{r}(t)}{dt}=\gamma_{h}\cdot H_{i}(t)-\mu_{h}\cdot H_{r}(t)\;$$ (1)

where time *t* is measured in weeks; *M*(*t*) = *M*_*s*_(*t*)  +  *M*_*e*_(*t*)  +  *M*_*i*_(*t*) represents the mosquito population; and *H* = *H*_*s*_(*t*)  +  *H*_*e*_(*t*)  +  *H*_*i*_(*t*)  +  *H*_*r*_(*t*) represents the human population. We add a new state variable $H_{i,total}(t)=\sum_{t\leq T}\theta_{h}$ ⋅ *H*_*e*_(*t*) to represent the cumulative dengue cases without accounting for recoveries. This is because the available data only includes newly exposed dengue cases, and the average period recovery for this population was unknown a priori, therefore, it is not possible to determine the number of infectious individuals for any given time. Table 1 represents the epidemiological description of each parameter in the compartmental model.

**Table 1:** Description of epidemiological parameters in the compartmental model.

Parameter	Epidemiological Meaning
$\beta_{h}$	Transmission rate from mosquito to human
$\beta_{m}$	Transmission rate from human to mosquito
$\gamma_{h}$	Recovery rate
*λ*	Mosquito recruitment
$\mu_{m}$	Mosquito mortality rate
$\mu_{h}$	Birth and mortality rate of human population
$\theta_{h}$	Transition rate from exposed to infectious humans
$\theta_{m}$	Transition rate from exposed to infectious mosquitoes

### 2.2 Basic reproductive number (*R*_0_)

The basic reproductive number in Eq (2), known as *R*_0_ (pronounced “R zero" or “R naught"), is a key indicator in the study of epidemics. It serves as a critical threshold parameter where values that exceed 1 indicate a potential for epidemic spread, while values below 1 suggest that the infection will eventually die out [23]. Researchers use the next generation matrix approach (NGM) [24] to determine *R*_0_, under the assumption that the population is initially free of infection (*H*_*i*_(0) = 0). The square root reflects the biological reality that vector-borne diseases require two generations (one vector generation and one host generation) to maintain ongoing transmission. The numerator represents transmission potential rates through mosquito-human and human-mosquito transmission rates ($\beta_{h}$, $\beta_{m})$ and incubation rates $(\theta_{h}$, $\theta_{m}$). The denominator accounts for the loss process, natural mortality, and recovery, which reduce effective transmission.

$$R_{0}=\sqrt{\frac{\beta_{m}\beta_{h}\theta_{m}\theta_{h}}{\mu_{m}(\theta_{m}+\mu_{m})(\theta_{h}+\mu_{h})(\gamma_{h}+\mu_{h})}}$$ (2)

### 2.3 Parameter estimation

We consider two parameter estimation frameworks, namely MCMC and NPC.

#### 2.3.1 Markov Chain Monte Carlo (MCMC).

The MCMC framework defines parameter bounds for the ODEs, explores the parameter spaces via Latin-hypercube Monte Carlo sampling, and then runs multiple local least-square fits from those starting points, following [25]. Specifically, for each proposed parameter vector *α*, the underlying ODE system is numerically integrated to produce the discrete state variables *x*(*t*_*i*_) at the observation time *t*_*i*_. Here, $f(x(t_{i}),\alpha )$ denotes the ODE function that defines $\frac{d}{dt}x(t_{i})$ as in Eq (3)

$$\frac{d}{dt}x(t_{i})=f(x(t_{i}),\alpha ),\;x(0)=x_{0}$$ (3)

The component $h(x(t_{i}),\alpha )$ selects the cumulative infectious case variable from the state vector *x*(*t*_*i*_). The difference between the output of $h(x(t_{i}),\alpha )$ and the observed *y*_*i*_ is quantified using the least squares loss function Eq (4).

$$J=\frac{1}{n}\sum_{i=1}^{n}{\left(y_{i}-h(x(t_{i}),\alpha )\right)}^{2}$$ (4)

MCMC proposes new parameter vectors and accepts them with a Metropolis-Hastings algorithm based on the posterior ratio, so increases in posterior density are always accepted while decreases are sometimes accepted. After discarding burn-in and checking convergence across multiple chains, the accepted proposals approximate the posterior, from which summaries such as the mean, median, and credible intervals provide parameter estimates with uncertainty. Although MCMC is a rigorous method, it often involves long burn-in periods, frequent sampling rejections that can lower efficiency, and reliance on stochastic sampling techniques such as random walks, which increase computational cost.

#### 2.3.2 Neural parameter calibration (NPC).

In NPC framework from [19] (Algorithm 1), we train a NN $u_{\theta }$ to directly predict both initial conditions and epidemiological parameters for the ECM, achieving orders of magnitude faster inference than traditional MCMC. The network, which functions as $u_{\theta }$, has three hidden layers with 20 neurons each, and uses only the logarithmic normalized cumulative dengue cases at *t* = 0 as input. It then outputs 14 estimated values $\alpha =(H_{i}(0),M_{e}(0),H_{r}(0),H_{s}(0),H_{e}(0),M_{s}(0),M_{i}(0),\beta_{h},\beta_{m},\gamma_{h},\lambda ,\mu_{m},\theta_{h},\theta_{m})$ with an additional constant $\mu_{h}=0.0004$ specified a priori. The chosen NN architecture effectively balances learning capacity and computational efficiency, providing accurate prediction with low run times. Its outputs are split into the initial conditions ϕ(0) and the parameter set *δ* that serve as input to a high–order numerical solver (specifically a Dormand–Prince Shampine Runge–Kutta method of order 5) to integrate the ECM over *L* time steps. At each step, the solver yields output variables $\hat{\phi }(t)$, whose first component $\hat{H_{i,total}(t)}$ is compared to the normalized observed cumulative dengue cases *H*_*i*,*total*_(*t*) via MSE loss as in Eq (5),

$$J(\hat{H_{i,total}(t)},H_{i,total}(t))=\frac{1}{L}\sum_{t=1}^{L}{\left(\hat{H_{i,total}(t)}-H_{i,total}(t)\right)}^{2}$$ (5)

**Algorithm 1 Single training epoch for NPC in ODE system.**

Inputs:

$H_{i,total}(t)=(H_{i,total}(0),...,H_{i,total}(L-1))$ {Observable Cumulative Infectious Cases}

*B* = *L* {Batch Size}

$\alpha \leftarrow u_{\theta }(H_{i,total}(0))$

Partition *α* into state variables $\hat{\phi }(0)$ and parameter set *δ*, set $\mu_{h}=0.0004$

**for** *t* = 1 **to** *L* **do**

$\hat{\phi }(t)\leftarrow SolveODE(\hat{\phi }(t-1),\delta )$ {Runge-Kutta order 5}

**end for**

Extract $\hat{H_{i,total}(t)}$ from $\hat{\phi }(t)$

Compute $J(\hat{H_{i,total}(t)},H_{i,total}(t))$ as *MSE* loss

Compute gradient $\nabla_{\theta }J$

Update *θ* via backpropagation (automatic differentiation)

**Fig 3:**
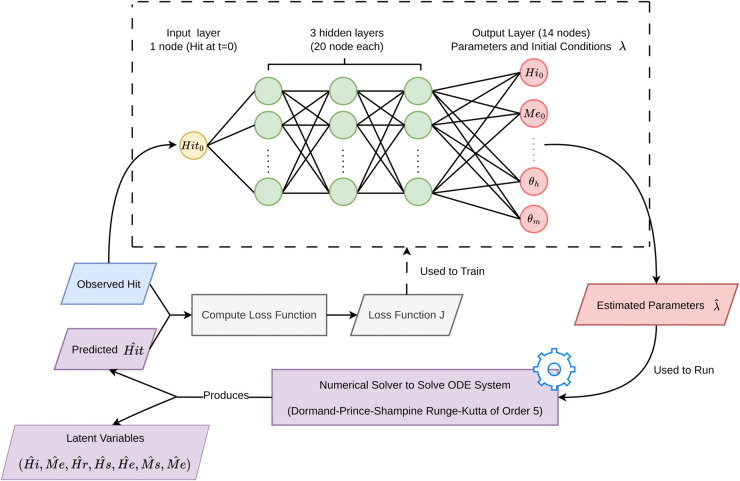
NPC workflow. In S1 Fig, we provide an illustration of the complete neural network model, including the input, hidden, and output layers.

Gradients $\nabla_{\theta }J$ are calculated by backpropagation through both the output from the numerical solver, enabling updates to the network weights *θ*, which are then used to produce the output *α* via feedforward. This forward-backward training loop iterates until convergence, after which the output $\hat{H_{i,total}(t)}$ is transformed back to the original scale by exp(x) − 1 for direct comparison with the observed data. The NPC workflow is summarized in Fig 3.

In traditional methods like MCMC, the posterior density is estimated by counting how often each point is sampled. However, in neural networks, the posterior is determined directly using the loss value *J* at each point, without relying on the sampling frequency. This means that we do not need to use rejection sampling or wait for a burn-in period because each calculation gives us the true likelihood immediately. Therefore, sampling the same point with the same information more than once is avoided, which makes the NN process much faster compared to the traditional MCMC. Furthermore, since the network movement is guided by the gradient $\nabla_{\theta }J$, it quickly focuses on the regions with the highest probability. This leads to a dense collection of samples around the most likely values, achieving faster and more accurate results compared to traditional sampling methods.

#### 2.3.3 Marginal posterior densities.

A marginal density distribution represents the probability distribution of a single parameter obtained by integrating the joint posterior distribution over all other parameters. This statistical technique enables visualization of the uncertainty associated with the estimation of each parameter individually, providing information on parameter identifiability and correlation structures within the model [20,25]. For the parameter vector $\alpha =(\alpha_{i},\alpha_{-i})$, the subscript _−*i*_ signifies all components of *α* except the component *ith* during the integration process. The marginal posterior density is mathematically expressed as Eq (6).

$$\rho (\alpha_{i}|H_{i,total}(t))=\int p(\alpha |H_{i,total}(t))d\alpha_{-i}$$ (6)

Where the integration is performed over the entire feasible domain of the remaining parameters $\alpha_{-i}$. The joint posterior distribution that appears in the integral is derived using the Bayes theorem as in Eq (7)

$$p(\alpha_{i}|H_{i,total}(t))=\rho (\alpha_{i}|H_{i,total}(t))*\pi^{0}(\alpha_{i})$$ (7)

Here, $p(\alpha_{i}|H_{i,total}(t))$ represents the likelihood function that captures the probability distribution of the parameters given the observational data. $\pi^{0}(\alpha_{i})$ denotes the joint prior distribution over all parameters. The only information we have a priori about the parameter values is that they are positive; hence, in the following, we always assume uniform priors on ${\mathbb{R}}_{+}$.

### 2.4 City datasets

**Fig 4:**
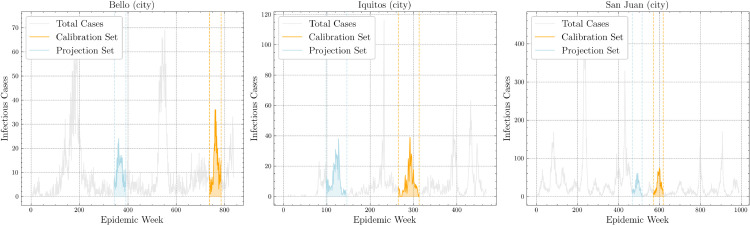
Selected calibration and projection dengue outbreaks from city data.

Time series of infectious dengue case were collected from laboratory-confirmed and clinically suspected case notifications: Iquitos (Peru) and San Juan (Puerto Rico) from National Oceanic and Atmospheric Administration (NOAA) [26]; and Bello (Colombia) from [25], with each dataset covering a 48-week epidemic window. Calibration periods were as follows: Bello, week 8 of 2014 to week 2 of 2015 (616 cases); Iquitos, week 6 of 2005 to week 1 of 2006 (426 cases); San Juan, week 51 of 2000 to week 46 of 2001 (1,492 cases). Projection periods were as follows: Bello, week 32 of 2006 to week 27 of 2007 (512 cases); Iquitos, week 47 of 2001 to week 42 of 2002 (515 cases); San Juan, week 52 of 1998 to week 47 of 1999 (1,158 cases). Fig 4 summarizes outbreak dynamics in city regions during calibration and projection.

### 2.5 Country datasets

**Fig 5:**
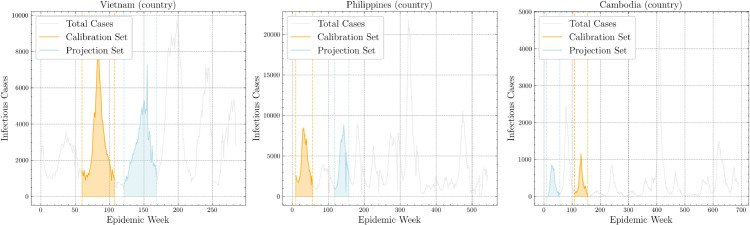
Selected calibration and projection dengue outbreaks from country data.

Time series of dengue cases for Southeast Asia were drawn from standardized public sources as described in [27]. Three high-incidence countries were analyzed over 48-week windows for calibration and projection. Calibration periods were as follows: Vietnam (week 9 of 2017 to week 4 of 2018, 142,854 cases), the Philippines (week 9 of 2013 to week 5 of 2014, 201,056 cases), Cambodia (week 5 of 2013 to week 48 of 2013, 16,889 cases). Projection periods were as follows: Vietnam (week 16 of 2018 to week 12 of 2019, 134,533 cases), the Philippines (week 13 of 2015 to week 9 of 2016, 199,811 cases), Cambodia (week 9 of 2011 to week 5 of 2012, 15,635 cases). Fig 5 summarizes the dynamics over these periods.

## 3 Experimental setup

We ran experiments on a 13th Gen Intel core i7-13620H CPU with 32GB RAM. Training used a batch size *B* = *L* (the outbreak-window length); the optimizer called Adam [28] with MSE between observed *H*_*i*,*total*_(*t*) and predicted $\hat{H_{i,total}(t)}$; a learning-rate scheduler with an initial value 5*e* − 4, linear warm-up over the first 20% of epochs followed by linear decay; and gradient clipping at 0.1. Weights were initialized following [29]; hidden layers use Sigmoid activations, and the output applied an absolute-value transform to enforce non-negative parameter estimates without functional output constraints. The model was trained for 2,000 epochs and run with 10 random seeds. During training, we tracked and aggregated the parameter ranges to define constrained priors for subsequent MCMC sampling.

Following [25], we implemented MCMC and ECM in MATLAB using the Symbolic Math Toolbox [30] and the GSUA-CSB Toolbox [31]. The ODE system was integrated with ODE45 (Dormand-Prince-Shampine Runge-Kutta method of order 5), to ensure comparability with the NPC solver. Parameters were estimated by minimizing MSE (Eq 4) via MATLAB’s lsqcurvefit (trust region reflective). Latin hypercube sampling (LHS) [32] generated 1,000 initializations, uniformly covering the parameter space. For each initialization, lsqcurvefit ran up to 4,000 model evaluations with default stopping tolerances *FunctionTolerance* = 1*e* − 6 and *StepTolerance* = 1*e* − 6. LHS provided diverse initializations, and local optimization refined them, outputting plausible parameter sets.

## 4 Results

### 4.1 Experiments on city data

**Fig 6:**
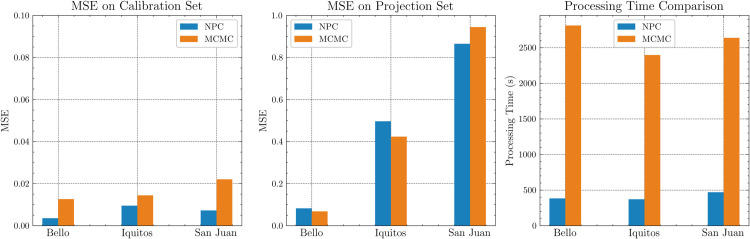
NPC vs MCMC accuracy and processing time on city data.

Across the three cities, NPC achieves significantly higher computational efficiency and accuracy comparable to MCMC. As shown in Table 2, NPC completes calibration approximately three orders of magnitude faster (seconds versus thousands of seconds; see Fig 6). For accuracy, NPC provides roughly three to four times reduction in MSE during calibration and similar MSE to MCMC during projection at all locations. The reported NPC MSE is averaged over multiple runs with different random seeds. These results indicate that NPC substantially reduces computational cost while maintaining predictive accuracy across diverse demographic contexts.

**Table 2:** Comparison of NPC and MCMC: MSE and processing time on city data.

City	Calibration MSE (on $H_{i,total}(t)$)		Projection MSE (on $H_{i,total}(t)$)		Running Time	
	NPC	MCMC	NPC	MCMC	NPC	MCMC
**Bello**	**0.00356**	0.01266	0.08236	**0.06822**	**383s**	2812.6453s
**Iquitos**	**0.00953**	0.01442	0.49630	**0.42315**	**371s**	2397.2065s
**San Juan**	**0.00725**	0.02206	**0.86532**	0.94443	**470s**	2638.1834s

**Fig 7:**
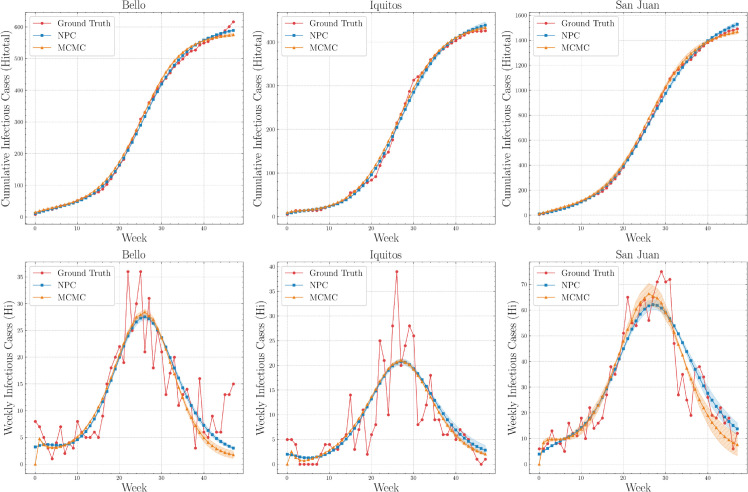
Comparison of NPC and MCMC accuracy for infectious (Hi) and cumulative infectious ($H_{i,total}(t)$) compartments on city data.

Fig 7 compares NPC and MCMC across dengue outbreaks in three cities. The blue and orange shaded bands denote standard deviation around the mean trajectory, computed across NPC networks with different random seeds and MCMC starting points, respectively. In the cumulative-incidence panels, NPC closely tracks the observations and performs similarly to MCMC across all cities, with small deviations during mid-epidemic growth, while MCMC appears to capture the total burden slightly better. In the weekly-incidence panels, NPC tracks key epidemic features, including peak timing and overall outbreak shape, despite data variability. Overall, NPC achieves accuracy comparable to that of MCMC in modeling the temporal dynamics of dengue transmission. Convergence of NPC training for city datasets, quantified by MSE over epochs, across random seeds is shown in S2 Fig.

**Fig 8:**
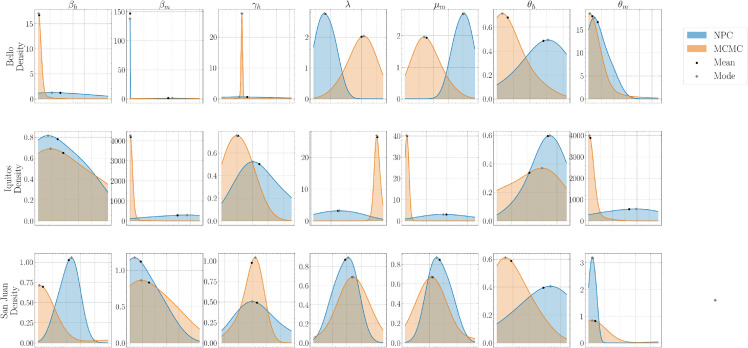
Comparison of NPC and MCMC for marginal posterior densities of ECM parameter set α on city data. The plot shows marginal posterior distributions for parameters in *α* after calibration in Bello, Iquitos, and San Juan. The x-axis gives parameter values and the y-axis gives posterior density. Narrow, sharp peaks indicate higher certainty, while broader shapes indicate greater uncertainty. Comparing these distributions across NPC and MCMC highlights differences in parameter uncertainty and estimation values.

Fig 8 compares marginal posterior distributions for parameters estimated using NPC (blue) and MCMC (orange) across the cities, with black dots and gray diamonds marking the posterior mean and mode, respectively. Across most parameters, both methods yield largely overlapping, unimodal posteriors with noticeable right-skew in several panels. Transmission and recovery rates, such as $\beta_{h}$, $\beta_{m}$ and $\gamma_{h}$ are highly concentrated and unimodal, whereas some vector-related parameters exhibit broader uncertainty. Notably, in the Bello datasets, *λ* and $\mu_{m}$ exhibit clear bimodality with two well-separated modes and minimal overlap between NPC and MCMC posteriors. Overall, central tendencies (mean and mode) are similar between methods, but uncertainty differs, with NPC typically producing narrower spreads while MCMC occasionally exhibits boundary-peaked densities.

**Fig 9:**
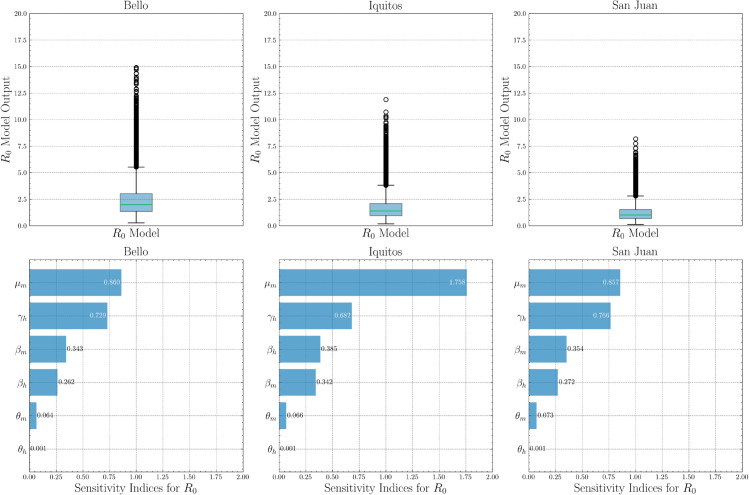
Sensitivity and uncertainty analysis for basic reproductive number $R_{0}$ on city data.

Fig 9 summarizes variability in *R*_0_ distribution and parameter sensitivities in Bello, Iquitos, and San Juan. The upper panels show broader *R*_0_ distribution in Bello (a median of 2.5 and outliers greater than 15), moderate spread in Iquitos (a median of approximately 2.0 and outliers of approximately 17.5), and the most constrained distribution in San Juan (a median of approximately 1.5 and outliers of approximately 12), indicating that most parameter sets produce moderate *R*_0_ distribution while some combinations provide extreme values. The lower panel shows consistent importance rankings: mosquito mortality $\mu_{m}$ is most influential (sensitivity about 0.86 in Bello, about 1.76 in Iquitos, about 0.86 in San Juan), while $\beta_{h}$, $\beta_{m}$, $\theta_{m}$, $\theta_{h}$, $\mu_{h}$ contribute modestly (approximately 0.00-0.39). These patterns suggest prioritizing vector control that reduces mosquito survival, with recovery-focused clinical interventions as a secondary level.

### 4.2 Experiments on country data

Building on the city-level results, the same framework is applied to national datasets to assess whether NPC’s advantages persist when scaling up dengue modeling from cities to countries.

**Fig 10:**
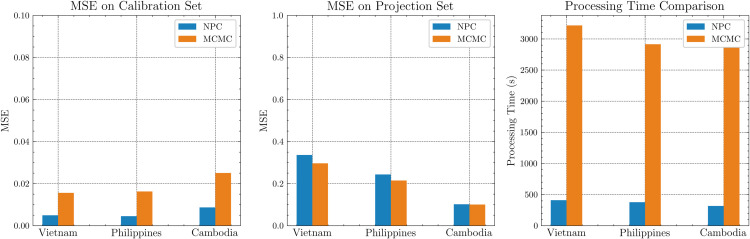
NPC vs MCMC accuracy and processing time on country data.

Across Vietnam, the Philippines, and Cambodia, NPC attains roughly 4 times lower calibration MSE and a similar projection MSE than MCMC, while calibrating orders of magnitude faster (seconds versus thousands of seconds), as summarized in Table 3 and visualized in Fig 10. These findings indicate that NPC scales from city- to country-level with substantial computational gains and comparable accuracy across diverse settings.

**Table 3:** Comparison of NPC and MCMC: MSE and Processing Time on Country Data

Country	Calibration MSE (on $H_{i,total}(t)$)		Projection MSE (on $H_{i,total}(t)$)		Running Time	
	NPC	MCMC	NPC	MCMC	NPC	MCMC
**Vietnam**	**0.00495**	0.01561	0.33628	**0.29672**	**410s**	3217.0232s
**Philippines**	**0.00449**	0.01625	0.24338	**0.21467**	**377s**	2915.1864s
**Cambodia**	**0.00871**	0.02505	0.10160	**0.10018**	**317s**	2864.2839s

**Fig 11:**
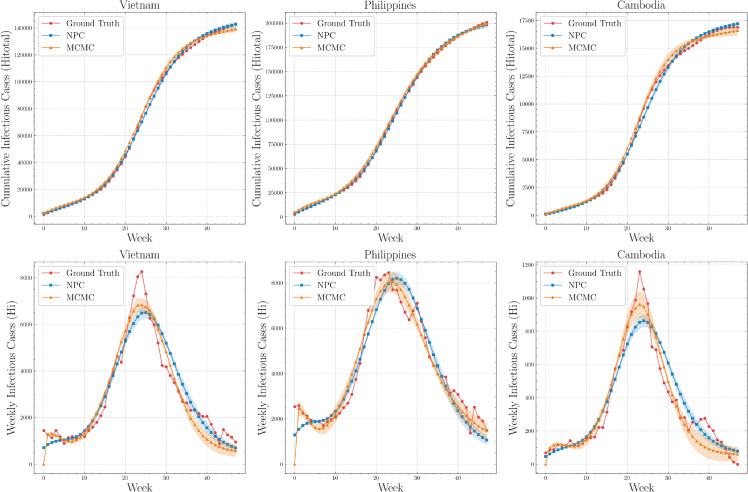
Comparison of NPC and MCMC accuracy for infectious (Hi) and cumulative infectious ($H_{i,total}(t)$) compartments on country data.

Fig 11 compares NPC and MCMC across Vietnam, the Philippines, and Cambodia. In the cumulative-incidence panels, NPC aligns closely with MCMC and the observations over the a 48-week window, with only minor deviations during mid-epidemic growth. In the weekly-incidence panels, NPC captures overall outbreak dynamics, while MCMC appears to better capture peak timing and magnitude. These country-level results are consistent with the city-level findings. Convergence of NPC training for country datasets, quantified by MSE over epochs, across random seeds is shown in S3 Fig.

**Fig 12:**
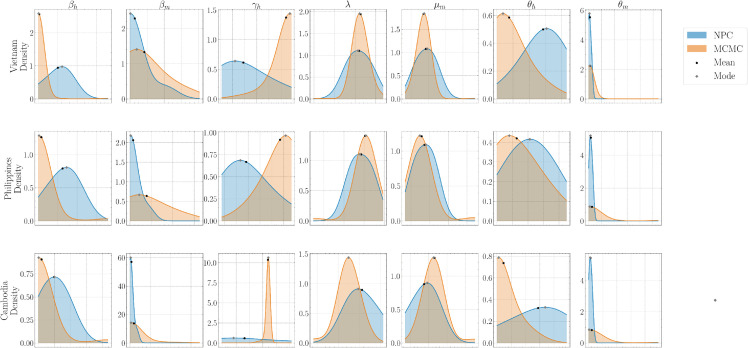
Comparison of NPC and MCMC for marginal posterior densities of ECM parameter set α on country data. The plot shows marginal posterior distributions for parameters in *α* after calibration in Vietnam, the Philippines, and Cambodia. The x-axis gives parameter values and the y-axis gives posterior density. Narrow, sharp peaks indicate higher certainty, while broader shapes indicate greater uncertainty. Comparing these distributions across NPC and MCMC highlights differences in parameter uncertainty and estimation values.

Fig 12 presents marginal posterior densities for each parameter from the national datasets (Vietnam, the Philippines, and Cambodia), with NPC in blue and MCMC in orange; black dots and gray diamonds mark the posterior mean and mode, respectively. Across parameters, both methods yield largely unimodal, substantially overlapping densities, with noticeable right-skew in several panels. For the transmission and recovery parameters $\beta_{h}$, $\beta_{m}$ and $\gamma_{h}$, the MCMC densities are typically more concentrated than NPC, particularly in Vietnam and Cambodia, while NPC shows broader spreads. The rate parameters *λ* and $\mu_{m}$ are unimodal across countries; MCMC is generally tighter and slightly right-shifted relative to NPC, indicating smaller posterior variance under MCMC. For $\theta_{m}$, the mass often concentrates near the lower boundary, with the sharper boundary peak alternating between methods across countries (sharper under MCMC in Vietnam, and under NPC in the Philippines and Cambodia). Overall, the national-scale results indicate that MCMC provides tighter uncertainty intervals than NPC.

**Fig 13:**
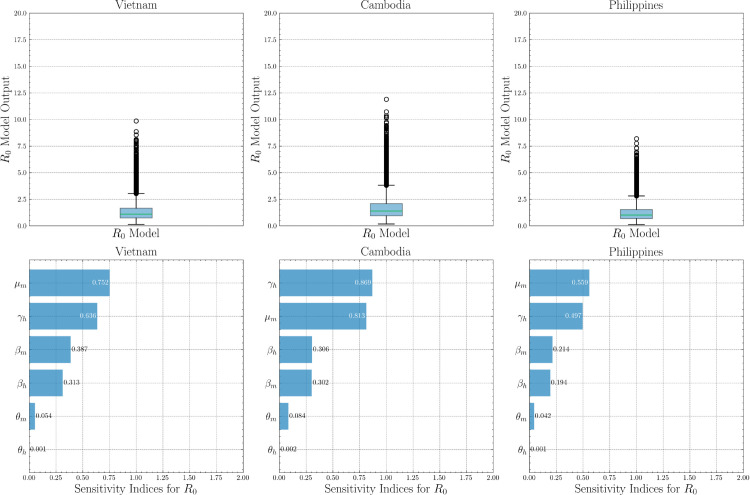
Sensitivity and uncertainty analysis for basic reproductive number $R_{0}$ on country data.

Fig 13 presents the uncertainty (box plots) and sensitivity results for *R*_0_ in Vietnam, the Philippines, and Cambodia. The *R*_0_ distributions are centered near or just above 1, with most values between 0.5 and 2, and with high outliers up to 10 (Vietnam), 8 (Philippines), and 12 (Cambodia), indicating potential for extreme transmission under certain parameter combinations. Sensitivity results identify mosquito mortality $\mu_{m}$ as most influential in Vietnam and the Philippines (indices 0.75 and 0.56), with human recovery $\gamma_{h}$ next; in Cambodia, $\gamma_{h}$ and $\mu_{m}$ are co-dominant (0.87 and 0.81). Other parameters, including $\beta_{h}$, $\beta_{m}$, and $\theta_{m}$, show moderate to low influence across countries. These patterns support prioritizing vector control while tailoring interventions to local drivers, including recovery dynamics.

## 5 Discussion

We used the ECM, consisting of human and mosquito components, formulated as seven equations, to model dengue transmission dynamics. We trained 10 independently initialized networks, with 3 hidden layers, 20 neurons each, for 2,000 epochs to estimate posterior distributions for parameters. We used MCMC to sample the posterior distributions of the same model parameters by defining priors and bounds using LHS, numerically integrating the ODE system for each proposal, and applying the Metropolis–Hastings algorithm across multiple chains. We quantified parameter uncertainty of NPC and MCMC’s and analyzed the sensitivity of the basic reproductive number. We validated the performance and generalizability of both frameworks on six heterogeneous dengue datasets: urban cases from Bello, Iquitos, and San Juan, and national cases from Vietnam, the Philippines, and Cambodia.

The analysis showed that, compared to MCMC, NPC achieves substantial speedups of approximately 7 times on city-scale datasets and 8 times on country-scale datasets over traditional MCMC, while being equally accurate with approximately 10% reduction in MSE on average for city data, and comparable accuracy for country data. Moreover, NPC yields consistently narrower posterior distributions and tighter uncertainty bounds than the broader MCMC posteriors. Sensitivity analysis of the basic reproductive number *R*_0_ reveals that mosquito mortality ($\mu_{m}$), human recovery ($\gamma_{h}$), mosquito-to-human transmission ($\beta_{m}$), and human-to-mosquito transmission ($\beta_{h}$) are the most influential parameters, with traditional MCMC also classifying these parameters $\beta_{m}$, $\beta_{h}$, $\gamma_{h}$, and $\mu_{m}$ as most influential [25]. The experimental results, consistent across diverse epidemiological locations in the Americas and Southeast Asia, demonstrate the generalizability of NPC, in particular when combined with ECM for dengue fever modeling and analysis.

However, our work still has limitations. The ECM only has human and mosquito components and did not consider climate and weather factors such as temperature, humidity, and precipitation, which are important to mosquito breeding [6]. Besides, NPC may suffer from overfitting or bias, necessitating careful design of network architectures and hyperparameter tuning. Future work should prioritize the integration of temperature, humidity, and precipitation into ECM and the use of NPC to jointly calibrate epidemiological, biological, and meteorological parameters and initial conditions.

## Supporting information

S1 FigIllustration of the neural networkvisualizes a deep neural network with three hidden layers. Here, the inputs (*H*_*i*,*total*_(0) shown in yellow) and the bias term (*x*_0_ shown in blue) pass through interconnected hidden layers, where edges visualize the weight matrices ${𝕎}_{i}$ and bias. The hidden units (green) process the activations to produce the output of the initial conditions and epidemiological parameters.(TIFF)

S2 Fig MSE loss convergence during NPC training with different random seeds on city data. (TIFF)

S3 Fig MSE loss convergence during NPC training with different random seeds on country data. (TIFF)
